# A Case of Compound Fracture of Both Tibia and Fibula, with a Few Practical Remarks

**Published:** 1844-11-16

**Authors:** Richard King

**Affiliations:** Surgeon


### A CASE OF COMPOUND FRACTURE OF BOTH TIBIA AND FIBULA, WITH A FEW PRACTICAL REMARKS.

By Richard King, Surgeon.

This case occurred in a boy, ætat. 14, of a healthy constitution. The accident happened as follows:— He was riding on horseback along the road, when another little boy came behind the horse, and gave it a blow with a stick, which caused the horse to plunge forward. The boy was thrown with great violence; and, on endeavouring to arise, he found he was unable to do so. He was, accordingly, brought home, a distance of one mile. I was sent for at 6 o'clock p. m., half an hour after the accident occurred; on my arrival at the house, I found the boy labouring under great pain, together with the shock caused by the injury, and some, though not much, loss of blood. On cutting off the shoe, and removing the stocking, I found the tibia and fibula driven out a little above the internal malleolus. The tibia was broken off at the epiphysis, and the fibula about two inches above the external malleolus; the wound, which was perpendicular, and the length of which was three inches, together with a transverse wound of two inches, corresponded to the lower extremity of the horizontal one; both bones protruded about four inches. On examining the wound, and passing my finger behind the protruding bones, there was a large cavity filled with coagulated blood, which was removed with a syringe, in order to see if any of the principal vessels were divided. The accident appeared of so serious a nature, as at first, to lead me to propose amputation to his parents, as the only means of saving his life (and to which they readily consented;) but, on considering the boy's age, and his being of a good constitution, it was determined on giving him a chance of saving his limb. With the kind assistance of Drs. Hunt and Booth, I commenced by first endeavouring to reduce the protruded bones; but, after many ineffectual attempts, I abandoned it, and carefully removed two inches of the bones, and a second time thought to reduce the remaining portion, in which I also failed, until the longitudinal wound was enlarged an inch and a half; they then readily admitted of reduction; the wound was brought together by four points of suture, and a roller applied; an anodyne draught was given, with cold evaporating lotions, externally, to the part. (The boy suffered very little during the operation.) There are a few important observations connected with this injury, which I will give in detail, after thus briefly sketching down the case. No bad symptoms ever set in all through; there was little or no suppuration; no exfoliation. The wound healed kindly; and I may mention the position which seemed most favourable,viz.; laid on its external surface, the leg semi-flexed on the thigh. The boy is now, three months after the accident, able to walk, with only the assistance of a stick; he has perfect use of the ankle-joint. There is a slight degree of shortening, which corresponds to the portion of bones removed; there is also thickening about the joint, but which is gradually lessening.

The remarks which I would wish to make, and deem of practical importance, both in a physiological and surgical point of view, might be divided into two sections:—First, as regards the injury; and secondly, the treatment to be adopted in such cases. We know, from experience, that injuries so approximated to a joint, and one of such magnitude, as the ankle, may be attended by the most serious consequences, particularly if the surgeon endeavour to save the limb. In this case, the tibia was broken off at its junction with the epiphyses, leaving its articular portion in connection with the joint, and driven out for the space of four inches through a lacerated wound in the soft parts; the fibula also protruded; its extremity was considerably spiculated; the foot was very much everted, and the bones protruded below its plantar surface; therefore, it was impossible to examine what extent of injury the joint had suffered; but, from the nature of the accident, it was deemed sufficient to have caused more or less injury. Secondly, as to the treatment of these injuries. The difficulty arising to this remark, is, to determine whether the limb ought to be saved, or amputation be performed, as soon as the patient recovers from the shock. This case, from its successful issue, and from the little disturbance which occurred, would induce a surgeon (in similar cases) to propose saving the limb; but I believe, in the majority of cases where such injury occurs, amputation is preferable.—*London Lancet.*

